# Ontogeny of RORγt^+^ cells in the intestine of newborns and its role in the development of experimental necrotizing enterocolitis

**DOI:** 10.1186/s13578-021-00739-6

**Published:** 2022-01-04

**Authors:** Xiuhao Zhao, Wenhua Liang, Yonghui Wang, Ruirong Yi, Lingjie Luo, Weifang Wang, Nannan Sun, Mingcheng Yu, Weijue Xu, Qingfeng Sheng, Li Lu, Jianfeng Pang, Zhibao Lv, Feng Wang

**Affiliations:** 1 Department of Gastrointestinal Surgery, Shanghai East Hospital, Tongji University School of Medicine, Shanghai, 200120 China; 2 Department of General Surgery, Shanghai Children’s Hospital, Shanghai Jiao Tong University, 355 Luding Road, Putuo, Shanghai, China; 3 Research Center of Translational Medicine, Shanghai Children’s Hospital, Shanghai Institute of Immunology, State Key Laboratory of Oncogenes and Related Genes, Shanghai Jiao Tong University School of Medicine, Shanghai, China; 4 Department of Medicinal Chemistry, School of Pharmacy, Fudan University, Shanghai, China; 5 Shanghai Institute of Immunology and Department of Immunology and Microbiology, Shanghai Jiao Tong University School of Medicine, 280 South of Chongqing Road, Huangpu, Shanghai, China

**Keywords:** Neonate, Necrotizing enterocolitis, Intestinal immune, RORγt

## Abstract

**Background:**

Neonates possess an immature and plastic immune system, which is a major cause of some diseases in newborns. Necrotizing enterocolitis (NEC) is a severe and devastating intestinal disease that typically affects premature infants. However, the development of intestinal immune cells in neonates and their roles in the pathological process of NEC have not been elucidated.

**Results:**

We examined the ontogeny of intestinal lamina propria lymphocytes in the early life of mice and found a high percentage of RORγt^+^ cells (containing inflammatory Th17 and ILC3 populations) during the first week of life. Importantly, the proportion of RORγt^+^ cells of intestinal lamina propria further increased in both NEC mice and patients tissue than the control. Furthermore, the application of GSK805, a specific antagonist of RORγt, inhibited IL-17A release and ameliorated NEC severity.

**Conclusions:**

Our data reveal the high proportion of RORγt^+^ cells in newborn mice may directly contribute to the development of NEC.

**Supplementary Information:**

The online version contains supplementary material available at 10.1186/s13578-021-00739-6.

## Background

Necrotizing enterocolitis (NEC) is a life-threatening disease that affects approximately 7% of infants with a birth weight under 1500 g [1]. The estimated mortality rate of NEC ranges from 20 to 30%, and a large proportion of NEC survivors remain at significant risk for short bowel syndrome and neurodevelopmental impairment [2–4]. Although the definitive causes of NEC remain unknown, prematurity is a recognized independent predictor because NEC occurrence increases with decreasing of gestational age and birth weight [5]. The other contributing factors include formula feeding, intestinal ischemia–reperfusion injury, dysbacteria, and excessive immune response [6]. Because of the complicated pathogenesis, there are no specific treatment strategies that effectively alter the outcome, and surgical approaches are the last resort but remain controversial. Recent studies suggest that human milk feeding and probiotics may prevent the development of NEC [7, 8], whereas the conclusions are also debatable [9].

Neonates possess a developing and plastic immune system, which is susceptible to different kinds of pathogenic factor invasion. Following birth, neonatal intestines have to encounter mass new antigens and stimuli, which increase the vulnerability of neonates to both infectious and non-infectious diseases [10]. Especially in preterm and very low birth weight infants, the intestinal barrier and immune system are immature, which predispose them to have risk of subjecting to late-on sepsis or NEC [11]. Studies have revealed that Toll-like receptor 4 (TLR4), a receptor for bacterial endotoxin, is expressed at a higher level on the intestinal epithelium of the premature human and mouse gut [12–14]. The excessive activation of TLR4 by lipopolysaccharide (LPS) leads to an infiltration of CD4^+^ T lymphocytes, and predispose the populations to pro-inflammatory type 17 T helper (Th17) cells, which is required for the development of NEC [15]. Meanwhile, a few studies have demonstrated that, compared with controls, intraepithelial γδ T lymphocytes and T regulatory cells (Treg) of lamina propria decreased significantly in surgical NEC specimens [11, 16–18]. The imbalance of Th17/Treg induces the increases of proinflammatory cytokines such as tumor necrosis factor (TNF), interleukin (IL)-1β, IL-6, IL-17, and IL-18, and the decreases of anti-inflammatory mediators such as IL-10 and transforming growth factor (TGF)β. The excessive inflammatory response triggers the vicious cycle that exacerbates tissue injury and necrosis of intestine, resulting in NEC development [19].

Although the phenomenon of pathogenesis is revealed gradually, the causative factors of NEC in preterm neonates remain poorly understood. Little is known about the state of intestinal immune system before NEC development. The orphan nuclear receptor retinoid-related orphan receptor γt (RORγt) is a key transcription factor that orchestrates the differentiation of immune cells [20] and induces the formation of lymph nodes and Peyer’s patches [21, 22]. In gut, RORγt^+^ cells include not only Th17 cells, but also several innate immune cells such as lymphoid tissue inducer cells (LTi), innate lymphoid cells (ILCs), γδ T cells, and natural killer (NK) cells [23]. After birth, LTi cells firstly cluster into cryptopatches of the intestinal lamina propria, accompanied with ILCs and RORγt^+^ T cells expand within the lamina propria [24]. Th17 cells are potent inducers of intestinal inflammation and have been implicated in the pathogenesis of inflammatory bowel disease (IBD) [25] and NEC [15]. However, the link between the ontogeny of RORγt and NEC development has not been revealed. In this study, we found that the high proportion of RORγt^+^ cells during the first few days of life, which might contribute to intestinal inflammation in neonates. Meanwhile, in NEC patients or mice model, the RORγt^+^ cells of intestinal lamina propria increased significantly than the healthy control. Furthermore, when NEC mice were treated with GSK805, the specific antagonist of RORγt, the intestinal lesions were improved visibly. In conclusion, we revealed that the high proportion of RORγt^+^ cells in neonatal intestine might be accountable for NEC development.

## Results

### Ontogeny of RORγt^+^ cells in intestinal lamina propria of neonatal mice

**Fig. 1:**
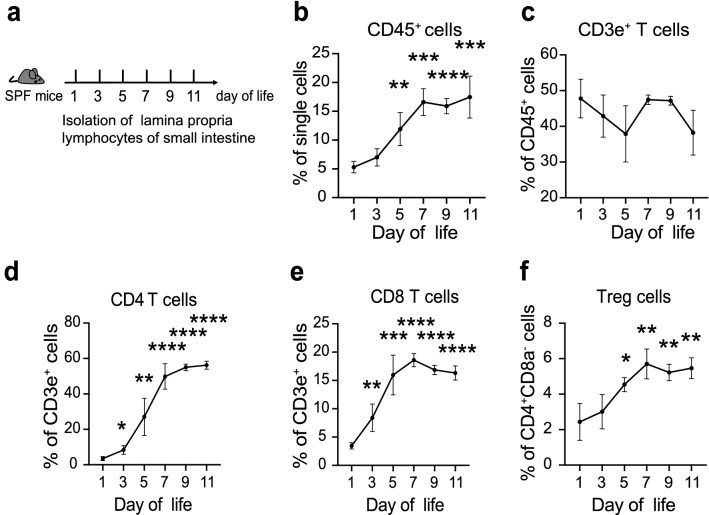
Dynamic changes of T cells in lamina propria of small intestine. **a**. Diagram of the experimental design. **b.** The changes tendency of leucocytes (CD45^+^ cells). **c**–**f**. The variations of CD3e^+^ T cells (**c**), CD4 T cells (**d**), CD8 T cells (**e**), and Treg cells (**f**) along with time. Three independent experiments were performed, n = 4–5 per group. Data were shown as mean values ± SD. Statistical analyses were performed with Student’s two-tailed unpaired *t*-test. *Compared with day 1. *p < 0.05; **p < 0.01; ***p < 0.001; ****p < 0.0001

The ontogeny of intestinal immune cells was investigated in normal neonatal mice to seek causes leading to intestinal disease in early life. The composition of immune cells within the lamina propria of small intestine was assessed throughout the first few days of life (Fig. 1a, Additional file 1: Fig. S1). Firstly, we noticed that the proportion of leukocytes (CD45^+^ cells) increased rapidly in the first 5 days, and kept stable after day 7 (Fig. 1b). However, the percentages of CD3e^+^ T cells vary dramatically during early life, which indicated the immature state of adaptive immune in the neonatal intestine (Fig. 1c). The average percentages of CD4 or CD8 T cells were below 5% at day 1 (Fig. 1d, e). The frequency of CD4 T cells climbed rapidly from day 3 to day 7, and remained over 50% after day 9. While, the population of CD8 T cells reached a peak of over 15% at day 7 and then decreased progressively (Fig. 1e). The percentages of CD4 and CD8 T cells at day 7 were consistent with the results in lamina propria of human infant intestines [26]. Whereas, the ontogeny trends of CD4 and CD8 T cells different from the study by Dingle et al. [17] showed the average percentage of CD4 T cells remained steady below 15%, and CD8 T cells kept over 75% in the terminal ileum since they measured total cellular content within the intestines in neonatal rats. Meanwhile, we found the percentages of CD4^+^Foxp3^+^ Treg cells progressively increased during the first week, then remained stable till to day 11 (Fig. 1f). These results characterized the ontogeny of intestinal adaptive immune cells in the early life of mice.

**Fig. 2:**
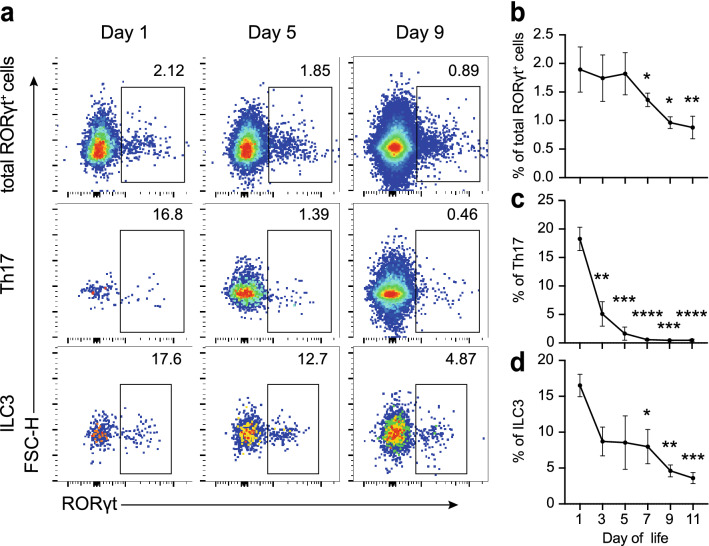
High percentages of RORγt^+^ cells during the first few days of life. **a**. Representative pseudocolor dot plots of total RORγt^+^ cells, Th17 and ILC3. **b**–**d** Dynamic changes of total RORγt^+^ cells, Th17 and ILC3. Th17 was gated on CD3e^+^CD4^+^CD8a^−^. ILC3 was gated on CD3e^−^CD8a^−^B220^−^LIN^−^CD90.2^+^. LIN includes CD11b and CD11c. Three independent experiments were performed, n = 4–5 per group. Data were shown as mean values ± SD. Statistical analyses were performed with Student’s two-tailed unpaired *t*-test. *Compared with day 1. *p < 0.05; **p < 0.01; ***p < 0.001; ****p < 0.0001

Interestingly, the highest percentage of RAR-related orphan receptor γ, isoform t (RORγt)-expressing cells among total CD45^+^ cells was observed in the first three days, which decreased to less than 1% after day 9 (Fig. 2a, b). Th17 and ILC3 cells are the two dominant populations that express the transcription factor RORγt in intestinal lamina propria, and they are responsible for various intestinal inflammation diseases [23]. Remarkably, our data showed that the average percentages of Th17 and ILC3 were more than 15% at day 1, and both of them decreased progressively (Fig. 2a, c, d). These findings prompted our hypothesis that the superabundant RORγt^+^ cells in the first few days of life might be resulting in the susceptibility to necrotizing enterocolitis induction.

### IL-17A^+^ RORγt^+^ cells accumulate in intestine of NEC mice

**Fig. 3:**
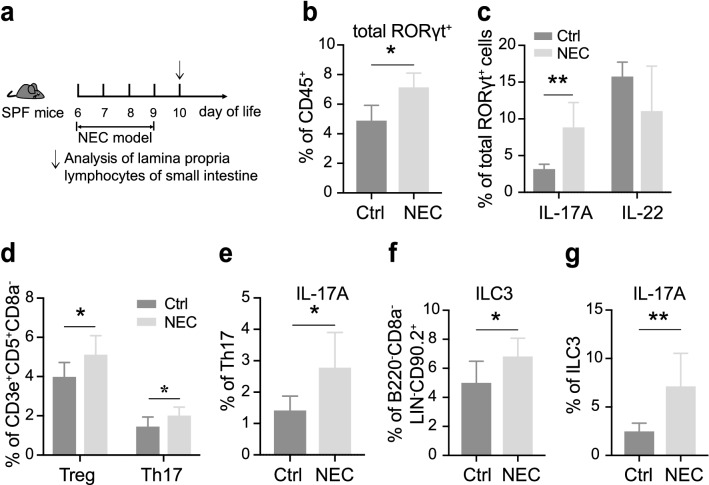
IL-17A^+^ RORγt^+^ cells increase in intestine of NEC mice. **a**. Diagram of the method to induce NEC. **b**, **c** Flow cytometric quantification of total RORγt^+^ cells and related cytokines (IL-17A and IL-22) in the lamina propria of mice with or without NEC. **d**–**g**. Flow cytometric quantification of Th17 and ILC3, as well as IL-17A expressed of them. Three independent experiments were performed, n = 4–5 per group. Data were shown as mean values ± SD. Statistical analyses were performed with Student’s two-tailed unpaired *t*-test. *p < 0.05; **p < 0.01

To examine the role of RORγt^+^ cells in NEC development, animal models were induced in neonatal mice as the method we used previously [27] (Fig. 3a). NEC was identified by the severity of weight loss and tissue impairment (Additional file 2: Fig. S2a, b). We first sought to compare the composition of lymphocytes within the lamina propria of small intestines in NEC and dam-fed control mice. Due to the most commonly affected site of NEC was ileum, we isolated the lymphocytes within lamina propria in small intestine and analyzed them with flow cytometry. Firstly, we observed that similar ratios of CD3e^+^ and CD4 T cells between the two groups and a reduction of CD8 T cells in NEC mice (Additional file 2: Fig. S2c, d). Not surprisingly, as shown in Fig. 3, the proportions of RORγt^+^ cells and IL-17A^+^ RORγt^+^ cells increased significantly in NEC when compared with controls (Fig. 3b, c). Furthermore, we found that the proportions of Th17 increased in NEC mice, and were accompanied by the increase of Treg (Fig. 3d). Meanwhile, IL-17A, the major inflammatory cytokine of Th17, was also elevated (Fig. 3e). In addition, we found that ILC3, the other dominant population expressed RORγt in intestinal lamina propria, as well as IL-17A^+^ ILC3 was also elevated when compared with control (Fig. 3f, g). Taken together, these findings suggest that the RORγt^+^ cells enriched intestinal environment predisposing to the induction of IL-17A lead to the development of NEC.

### T cells and RORγt^+^ cells increased in intestine of NEC patients

**Fig. 4:**
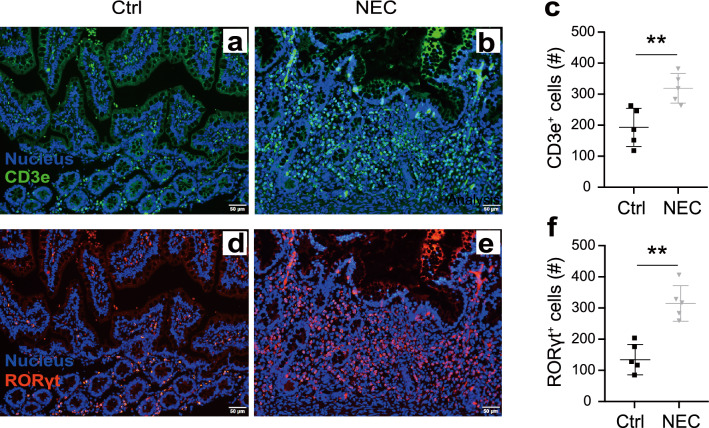
Changes of T cells and RORγt^+^ cells in NEC patients. Representative immunofluorescent staining pictures, and the statistical analyses of the count of CD3e^+^ T cells (**a**–**c**) and RORγt^+^ cells (**d**–**f**). n = 4 per group. Data were shown as mean values ± SD. Statistical analyses were performed with Student’s two-tailed unpaired *t*-test. **p < 0.01. Scale bars represent 50 μm. Ctrl, tissue from neonatal intestinal atresia patients. The Y-axis means total cell number in a visual field of one patient’s terminal ileum (**c**, **f**)

To examine whether the frequencies of RORγt^+^ cells were perturbed in NEC patients, we detected the composition of lymphocytes within the terminal ileum. As shown by immunofluorescent staining, the number of CD3e^+^ T cells in NEC patients increased notably when compared with controls (Fig. 4a–c). This is consistent with the previous study [15]. Meanwhile, we found a significant increase of RORγt^+^ cells in NEC patients as compared with controls (Fig. 4d–f). These data reminded us the influx of RORγt^+^ cells in the intestine is associated with the development of NEC in humans.

### RORγt antagonist (GSK805) ameliorates the severity of NEC by suppressing IL-17A

RORγt is an attractive therapeutic target for the treatment of IL-17-mediated inflammatory disease. Based on our previous results, we considered whether the RORγt antagonist could inhibit IL-17A expressed to prevent the development of NEC in mice. Several small-molecular-weight compounds, including TMP778, SR1001, digoxin, and GSK805 [28–30], have been identified to inhibit the function of RORγt protein and showed efficacy in models of autoimmunity disease. Among them, GSK805 presents more potent at inhibiting Th17 responses in vitro and ameliorating the severity of experimental autoimmune encephalomyelitis (EAE) by oral administration [30].

**Fig. 5:**
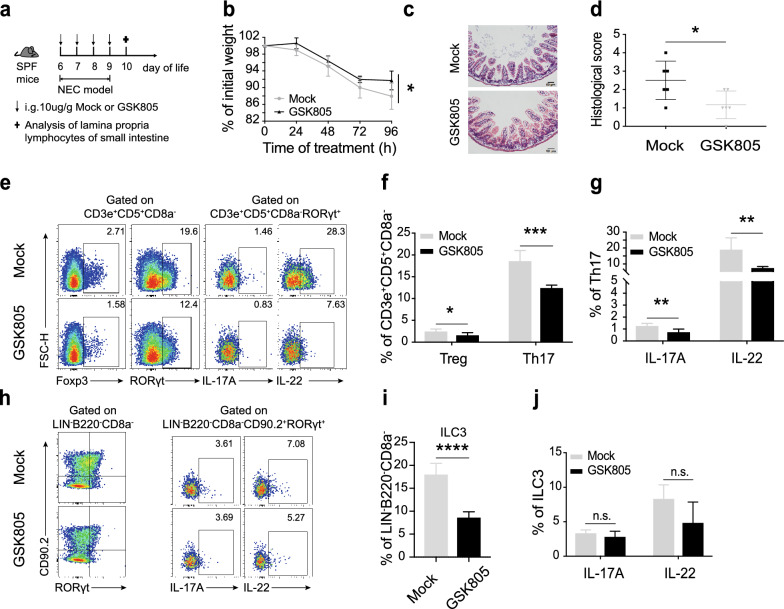
GSK805 alleviates intestinal inflammation of NEC mice by inhibiting IL-17A released. **a**. Scheme of the method of GSK805 treatment. Mice were treated with Mock or GSK805 (10 μg/g) orally. GSK805 was prepared with 1% DMSO in CMC-Na. **b**. Percentage of initial weight. **c**. Representative hematoxylin and eosin staining sections of the terminal ileum. **d**. Histopathological score measuring the severity of tissue lesions in the terminal ileum. **e**–**g**. Flow cytometric analysis and quantification of T cell subsets and related cytokines (IL-17A and IL-22) within lamina propria in small intestine. **h**–**j**. Flow cytometric analysis and quantification of ILC3 and related cytokines (IL-17A and IL-22). Four independent experiments were performed, n = 4–5 per group. Data were shown as mean values ± SD. Statistical analyses were performed with Student’s two-tailed unpaired *t*-test. *p < 0.05; **p < 0.01; ***p < 0.001; ****p < 0.0001. Scale bars represent 50 μm

We next examined the in vivo effects of GSK805 on NEC mice. We induced NEC in neonatal mice and treated them with GSK805 orally once a day (Fig. 5a). Compared with Mock (control), GSK805 treatment delayed weight loss and substantially reduced the intestinal tissue impairment demonstrated by hematoxylin and eosin staining and histopathological scores (Fig. 5b–d). Analysis of intestinal samples after 4 days of GSK805 treatment revealed that the drug had no effect on intestinal barrier (Additional file 3: Fig. S3a, b). However, GSK805 effectively reduced the infiltration of immune cells, including both Th17 and Treg cells, and suppressed the expression of IL-17A and IL-22 (Fig. 5e–g). Furthermore, we found that GSK805 significantly inhibits the expression of RORγt in ILCs, resulting in the decrease of ILC3 (Fig. 5h, i). Interestingly, GSK805 had no effect on cytokines expression from ILC3 (Fig. 5h, j), which might be associated with the selective targeting of GSK805 on Th17 and ILC3 [31, 32]. Taken together, these results demonstrate that the RORγt antagonist provides therapeutic potential by inhibiting Th17 responses.

## Discussion

With the prominent work of researchers over the last few decades, scientists have made considerable progress in understanding the pathogenesis of NEC. However, there has been very little focus on the state of immune cells within the intestine in newborns before the onset of this disease. Previous studies examining the postnatal ontogeny of lymphocytes in the intestine of human neonates show that CD3^+^, CD4^+^, CD8^+^, and Foxp3^+^ cells are present in the lamina propria of infants after birth [16]. However, they were unable to find a difference in the proportion or quantities of Treg between term and preterm samples by immunohistochemistry due to technical limitations. Later studies revealed a significant reduction of the proportion of Treg in the ileum of NEC patients [17, 26]. Recent research has characterized the pathoimmunology of NEC and found that the increase of type 3 innate lymphoid cells (ILC3) and deficiency in IL-37 have association with the development of NEC [33]. In the present study, we focused on the state of intestinal immunity before NEC happened and investigated the compositions of immune cells within the small intestine during the early period of neonatal mice. We found that the proportion of RORγt^+^ cells is very high in the first week of life. Moreover, IL-17A^+^RORγt^+^ cells accumulated in the small intestine of NEC mice which might be attributed to the disease. Furthermore, inhibiting the function of RORγt^+^ cells by GSK805 ameliorated the severity of NEC. These findings provide new insight into the pathomechanistic understanding and treatment of NEC.

The nuclear hormone receptor RORγt, as an inducer of the pro-inflammatory program in lymphocytes, plays a key role in the regulation of immune responses and the maintenance of immune homeostasis. During ontogeny of fetuses, RORγt is firstly expressed in lymphoid tissue inducer (LTi) cells, which determines the development of lymph nodes and Peyer’s patches [21, 34]. Shortly after birth, RORγt^+^ type 3 innate lymphoid cells (ILC3) and RORγt^+^ T cells expand within the intestinal lamina propria. RORγt^+^ T cells include subsets of γδT cells, invariant natural killer T (iNKT) cells, and Th17 cells [24, 35]. All RORγt^+^ subsets express IL-17, which have been implicated in multiple human chronic inflammatories and autoimmune diseases, including inflammatory bowel disease (IBD) [36], multiple sclerosis [37], rheumatoid arthritis [38], and psoriasis [39]. A recent study has demonstrated that the expression of intestinal IL-17A and IL-17 receptor IL-17RA are elevated during human and mouse NEC [15]. Meanwhile, the other study has shown the risk and outcome of NEC in preterm infants is positively associated with the increase in single nucleotide polymorphisms of IL-17F and IL23R [40]. Here, we firstly revealed that the proportion of RORγt^+^ cells within intestinal lamina propria was at a high level during the first few days of life. Moreover, IL-17A produced by RORγt^+^ cells (Th17 and ILC3) increased significantly in NEC mice compared with control. Therefore, it is plausible that the high level of RORγt^+^ cells within intestines places the newborns at high risk for intestinal inflammation, resulting in a susceptibility to NEC.

Therapeutic targeting of the Th17 cell pathway or blocking of IL-17A has been proved effective in several autoimmune diseases [41, 42], but ineffective in IBD [43–45]. Later researches ascribe the paradoxical effect to the protective functions for IL-17A and IL-22 in the intestine, mediated in part by ILC3 [46–48]. In recent years, inhibition of RORγt by small-molecular-weight compounds offers an alternative method for the treatment of autoimmune disease and intestinal inflammation [28, 29, 31, 49]. Among inhibitors of RORγt, orally available compound GSK805 presents more potent at inhibiting Th17 responses and ameliorating experimental autoimmune encephalomyelitis (EAE) [49]. GSK805 interacts physically with the putative ligand-binding domain of RORγt, but exert less pronounced effects on DNA binding than other inhibitors [49]. Meanwhile, GSK805 has been demonstrated to selectively reduce cytokine production from Th17 but not ILC3, for limiting intestinal inflammation [31]. Accordingly, we investigated the effectiveness of GSK805 in alleviating the severity of NEC. Not surprisingly, GSK805 treatment improved the survival and weight loss in NEC mice and reversed the histological lesion of the intestine. Notably, GSK805 selectively suppressed IL-17A release from Th17 but not ILC3, as discussed above, contributed to its role in ameliorating NEC.

## Conclusion

In summary, we characterized the immune state within intestinal lamina propria during the first few days of mouse life. Meanwhile, our results revealed the proportion of RORγt^+^ cells are at a high level shortly after birth. This might be associated with the development of NEC in newborns. Furthermore, we demonstrated that GSK805, as the selective inhibitor of RORγt, has the potential to mitigate the severity of NEC by inhibiting Th17 function. These results can be very instructive in understanding the pathological mechanism and exploring new effective therapeutic methods for NEC.

## Methods

### Mice

C57BL/6 neonatal mice were provided by Shanghai Ling Chang Biotech limited company. Mice were maintained under SPF conditions in the Animal Science Centre at the Shanghai Jiao Tong University School of Medicine. Animal experiments were approved by the Animal Care Committee of Children's Hospital of Shanghai and Shanghai Jiao Tong University School of Medicine.

### Reagents and antibodies

The drug GSK805 was synthesized and provided by Yonghui Wang’s laboratory. Collagenase VIII (C2139) and DNase I (DN25) were purchased from Sigma-Aldrich. Cell Stimulation Cocktail (plus protein transport inhibitors) (00-4975-93), Foxp3/Transcription Factor Staining Buffer Set (00-5523-00), Permeabilization Buffer (10×) (00-8333), anti-CD3e (45-0031-82), and anti-Foxp3 (25-5773-82) were provided by Thermo Fisher SCIENTIFIC. Anti-CD45 (30-F11), anti-CD4 (553051), anti-CD8a (557668), anti-CD90.2 (563008), anti-B220 (563892), and anti-RORγt (564722) were purchased from BD Bioscience. Anti-CD11b (101224) and anti-CD11c (117322) were purchased from Biolegend.

### Experimental design and NEC model

For analyzing the ontogeny of intestinal immune cells in the early life of mice, neonatal mice born at the appointed time were purchased directly from the company. Those mice were sacrificed on the same day for the experiment. For the other experiments, 6- to 7-day-old mice were randomly divided into the NEC + Mock group, NEC + GSK805 group, or control group. Mice in the control group were dam-fed without any other stimulus. NEC model was induced using a method as our previous study [27] modified from Jilling et al. [50]. Briefly, neonatal mice were fed with 50 μl of 33% Esbilac formula (Pet-Ag) by gavage every 4 h for 96 h. Meanwhile, newborns were subjected to hypoxia (99.9% N_2_ for 90 s) followed by cold stress (4 °C for 10 min) twice a day for 4 days. During the NEC modeling process, mice were treated with Mock or GSK805 (10 μg/g) by intragastric administration once a day for four days. All mice were weighed every morning and euthanized at 96 h for collecting intestinal samples.

### Histological analysis

Terminal ileum tissues were fixed with 4% paraformaldehyde, embedded in paraffin, and sectioned, then stained with H&E. NEC was evaluated based on histological changes of the terminal ileum according to a previously described scoring system [51]. The severity of NEC was classified as follows: intact villi were assigned a score of 0, sloughing of cells on villous tips received a score of 1, and mid-villous damage was scored as 2. An NEC score of 3 was recorded when villi were absent, but crypts were still readily detectable, and an NEC score of 4 was assigned in cases of a complete absence of epithelial structures and transmural necrosis. Scores were always determined based on the highest score observed in a specimen.

### Isolation of intestinal lamina propria lymphocytes

Intestinal lamina propria lymphocytes in newborns were isolated as our previous method with a modification [52]. In brief, the small intestine excluded duodenum was removed and cut longitudinally. After washing in PBS by a few sharp shakes, the tissue pieces were transferred into solution A (1 mM DTT, 30 mM EDTA, 10 mM HEPES) and incubated in a shaker at 37 °C for 10 min. Then, transferred the tissues into solution B (30 mM EDTA, 10 mM HEPES) and repeated as the previous step. After washing with complete RPMI 1640 medium, the tissues were cut into 1 mm^3^ piece and digested in RPMI 1640 containing 500 μg/ml collagenase VIII and 100 μg/ml DNase I at 37 °C for 50 min. Due to a relatively low number of lymphocytes in the neonatal intestine, it is not suitable for purifying the cells. Hence, we next washed the single-cell suspension with FACS buffer (0.5% BSA-PBS) and stained with flow antibodies.

### Flow cytometry

Intestinal cells were firstly incubated with cell stimulation mixture plus protein transport inhibitor (Thermo) in complete RPMI 1640 at 37 °C for 4 h. After stimulation, cells were stained with antibodies conjugated with the indicated fluorochrome for surface marker analysis. The intracellular expression levels of transcriptional factors and cytokines were detected as the manufacturer’s instructions of Foxp3/Transcription Factor Staining Buffer Set (Thermo). Flow cytometric analyses were performed using a LSRFortessa X-20 (BD Biosciences) and analyzed with FlowJo 10.4 software.

### Statistical analysis

Statistical analysis was performed with Student’s two-tailed unpaired *t*-test and one-way or two-way analysis of variance (ANOVA) followed by Welch’s or Mann–Whitney test. For weight change analyses, two-way ANOVA was used, with treatment as the main effect. All data are expressed as mean values ± SD. *p* < 0.05 was considered to be significant. Statistical analysis was performed by GraphPad Prism 8.0.

## Supplementary Information

**Additional file 1: Figure S1.** Gate strategy of flow cytometry analysis.The methods to analyze flow data with FlowJo software. LIN presents CD11b and CD11c.

**Additional file 2: Figure S2.** Confirmation of NEC model and the changes of T cells. **a**. Percentage of initial weight. **b**. Representative hematoxylin and eosin staining sections of the terminal ileum. **c** and **d**, Changes of CD3e^+^, CD4, CD8 T cells between Ctrl and NEC mice.

**Additional file 3: Figure S3.** Effect of GSK805 on intestinal barrier. **a**. Serum concentrations of FD4 in NEC mice treated with GSK805 or not. **b**. Representative images of tight junction proteins in small intestinal epithelial cells. FD4, FITC-dextran 4 kDa.

## Data Availability

Not applicable.

## Abbreviations

NEC: Necrotizing enterocolitis
RORγt: Retinoid-related orphan receptor γt
ILC: Innate lymphoid cells
LTi: Lymphoid tissue inducer cells
TNF: Tumor necrosis factor
TGF: Transforming growth factor
