# Supplementary figures and images for: Ontogeny of RORγt+ cells in the intestine of newborns and its role in the development of experimental necrotizing enterocolitis
Source: Cell Biosci. 2022 Jan 4;12:3. doi: 10.1186/s13578-021-00739-6 (PMC8725364; doi:10.1186/s13578-021-00739-6)

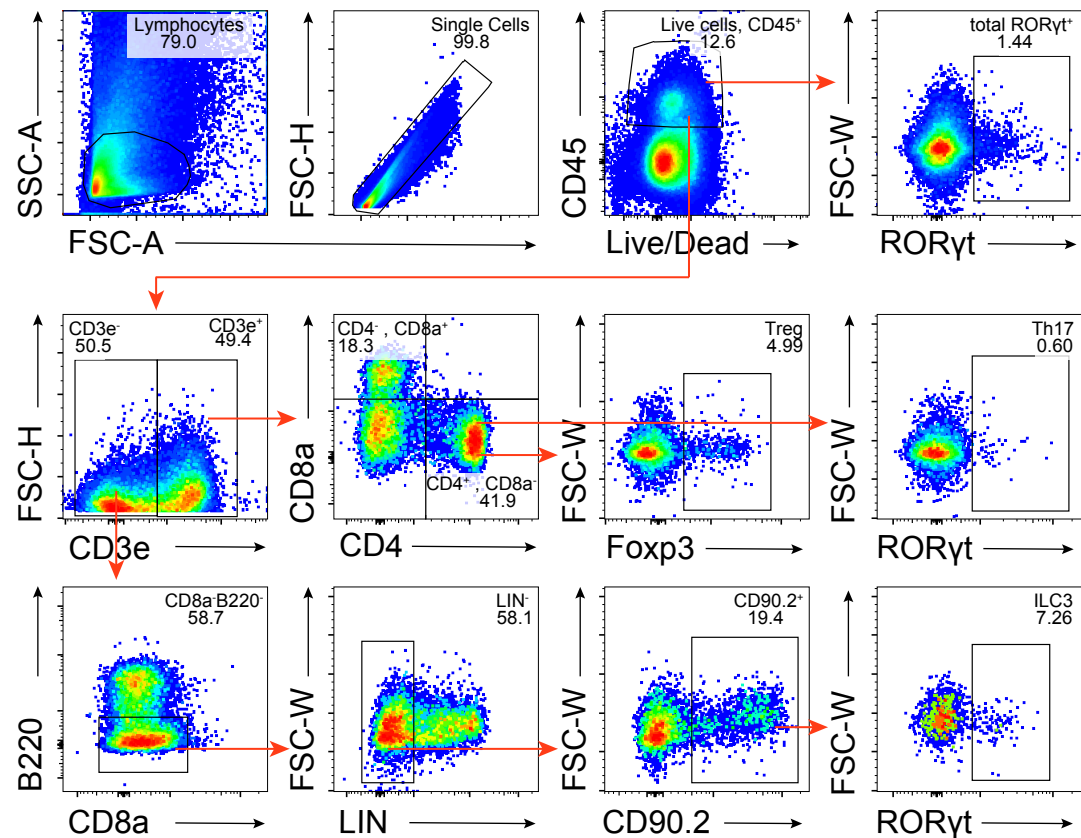

LIN: CD11b, CD11c

Supplement: Supplementary file 1 — Additional file 1: Figure S1. Gate strategy of flow cytometry analysis.The methods to analyze flow data with FlowJo software. LIN presents CD11b and CD11c. [file 13578_2021_739_MOESM1_ESM.pdf]

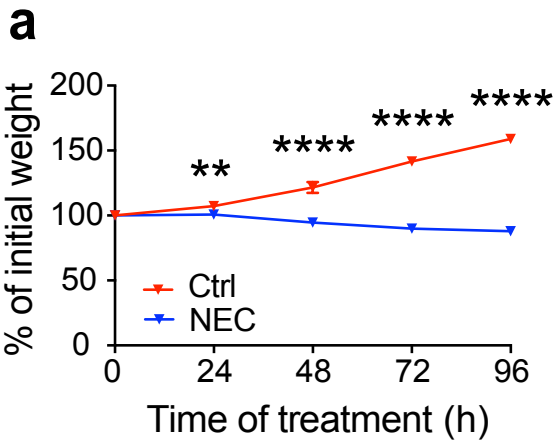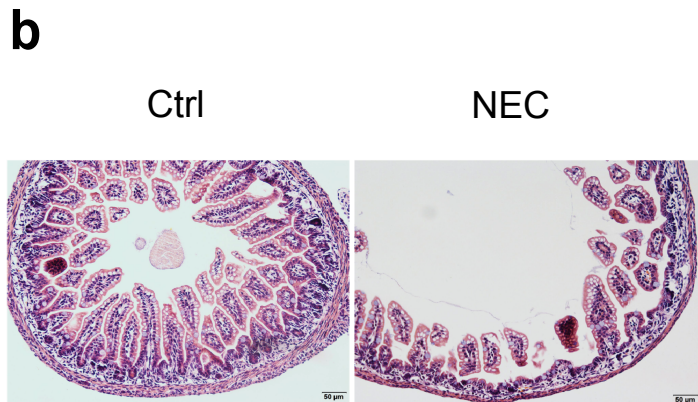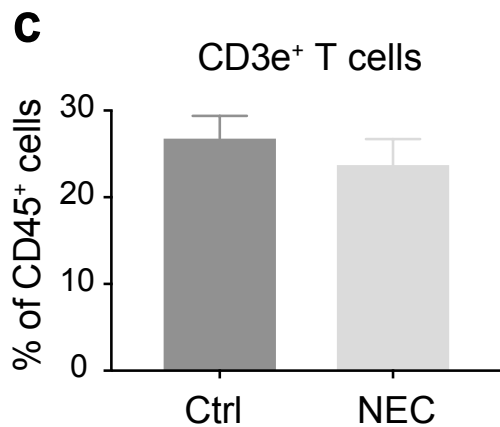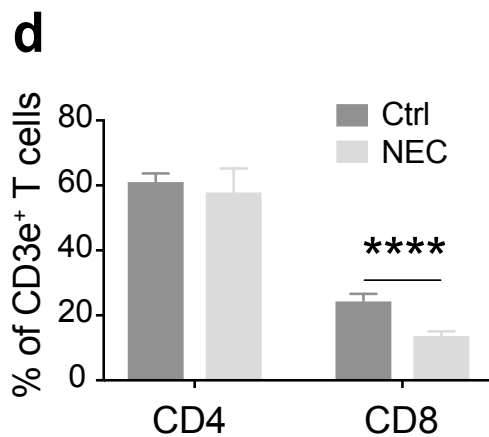

Supplement: Supplementary file 2 — Additional file 2: Figure S2. Confirmation of NEC model and the changes of T cells. a. Percentage of initial weight. b. Representative hematoxylin and eosin staining sections of the terminal ileum. c and d, Changes of CD3e+, CD4, CD8 T cells between Ctrl and NEC mice. [file 13578_2021_739_MOESM2_ESM.pdf]

**a**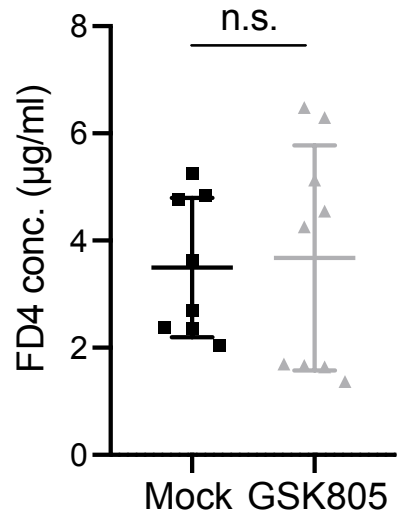**b**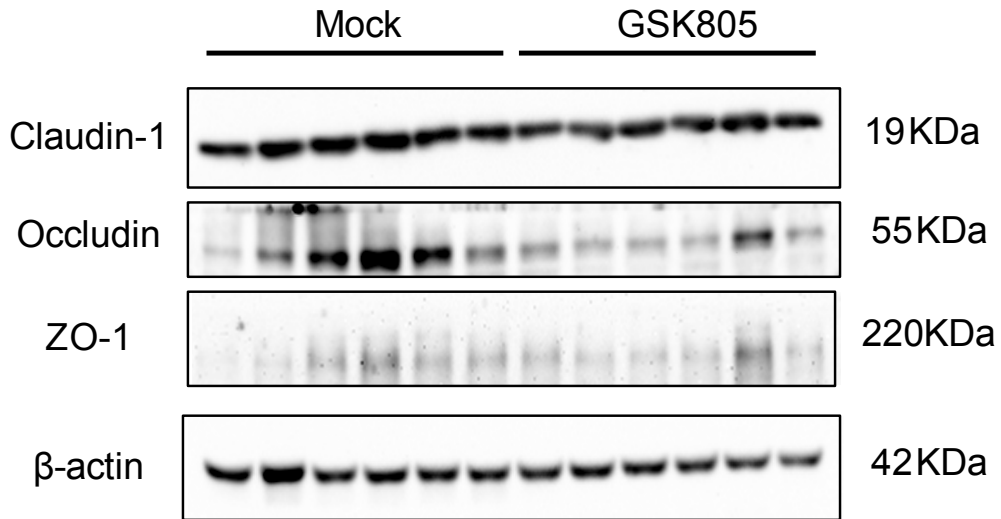

Supplement: Supplementary file 3 — Additional file 3: Figure S3. Effect of GSK805 on intestinal barrier. a. Serum concentrations of FD4 in NEC mice treated with GSK805 or not. b. Representative images of tight junction proteins in small intestinal epithelial cells. FD4, FITC-dextran 4 kDa. [file 13578_2021_739_MOESM3_ESM.pdf]
